# Milan PM1 Induces Adverse Effects on Mice Lungs and Cardiovascular System

**DOI:** 10.1155/2013/583513

**Published:** 2012-12-27

**Authors:** Francesca Farina, Giulio Sancini, Eleonora Longhin, Paride Mantecca, Marina Camatini, Paola Palestini

**Affiliations:** ^1^Department of Health Science, POLARIS Research Center, University of Milan-Bicocca, 48 Via Cadore, 20900 Monza, Italy; ^2^Department of Environmental Science, POLARIS Research Center, University of Milan-Bicocca, 1 piazza della Scienza, Milan 20126, Italy

## Abstract

Recent studies have suggested a link between inhaled particulate matter (PM) exposure and increased mortality and morbidity associated with cardiorespiratory diseases. Since the response to PM1 has not yet been deeply investigated, its impact on mice lungs and cardiovascular system is here examined. A repeated exposure to Milan PM1 was performed on BALB/c mice. The bronchoalveolar lavage fluid (BALf) and the lung parenchyma were screened for markers of inflammation (cell counts, tumor necrosis factor-**α** (TNF-**α**); macrophage inflammatory protein-2 (MIP-2); heme oxygenase-1 (HO-1); nuclear factor kappa-light-chain-enhancer of activated B cells p50 subunit (NF**κ**B-p50); inducible nitric oxide synthetase (iNOS); endothelial-selectin (E-selectin)), cytotoxicity (lactate dehydrogenase (LDH); alkaline phosphatase (ALP); heat shock protein 70 (Hsp70); caspase-8-p18), and a putative pro-carcinogenic marker (cytochrome 1B1 (Cyp1B1)). Heart tissue was tested for HO-1, caspase-8-p18, NF**κ**B-p50, iNOS, E-selectin, and myeloperoxidase (MPO); plasma was screened for markers of platelet activation and clot formation (soluble platelet-selectin (sP-selectin); fibrinogen; plasminogen activator inhibitor 1 (PAI-1)). PM1 triggers inflammation and cytotoxicity in lungs. A similar cytotoxic effect was observed on heart tissues, while plasma analyses suggest blood-endothelium interface activation. These data highlight the importance of lung inflammation in mediating adverse cardiovascular events following increase in ambient PM1 levels, providing evidences of a positive correlation between PM1 exposure and cardiovascular morbidity.

## 1. Introduction

Epidemiology studies have shown that increased levels of particulate matter (PM) in ambient air are associated with aggravation of respiratory diseases and cardiovascular function impairment. These adverse events have been correlated with exposure to fine PM (particles with aerodynamic diameter ≤2.5 *μ*m) [1], even if the pathophysiological mechanisms remain still unclear.

Lung PM penetration and clearance are size dependent: larger particles (greater than 10 *μ*m), deposited in the upper airways, are removed by the mucociliary clearance mechanism, while smaller particles (below 10 *μ*m) reach deeper the lungs and are only partially removed by alveolar macrophages (AMs) [2]. 

Following PM deposition in the lungs, AMs rapidly phagocytose particles and migrate towards the broncho-alveolar junction [3]. A large number of ultrafine particles (≤0.1 *μ*m in aerodynamic diameter), however, poses a substantial burden for the macrophage phagocytic system and results in increased number of particles coming in contact with the respiratory epithelium. Damage to the capillary endothelium and type I alveolar cells has been observed as one of the earliest events in lung toxicity mediated by particles, leading to neutrophils recruitment and triggering the onset of an acute inflammatory status [2, 4]. Moreover, PM contains transition metals able to generate reactive oxygen molecules which in turn exert a cytotoxic effect on lung cells [2].

Translocation of inhaled nanoparticles across the alveolar-blood barrier has been demonstrated in animal studies for a range of nanoparticles delivered by inhalation or instillation [5–7]. Convincing demonstration of translocation has been difficult to achieve in humans [8, 9]; however, given the deep penetration of nanoparticles into the alveoli and the close apposition of the alveolar wall and capillary network, such particle translocation seems plausible either as a naked particle or after ingestion by AMs.

Many authors proposed the hypothesis that fine particles inhalation provokes a low grade inflammatory response in the lung, causing an exacerbation of preexisting lung diseases. We previously reported [10] that a single intratracheal instillation of fine particles in mice stimulates mild lung inflammation. The present study extends these findings by showing that the repeated deposition of particulate matter in the lungs triggers the onset of systemic adverse events. 

Milan's particles concentration and its chemical composition have been widely examined [11, 12]. Despite the number and quality of chemical data, the biological effects produced on *in vivo* and *in vitro* systems have been only recently and partly investigated [4, 10, 13, 14]. In this paper we present and discuss pulmonary and cardiovascular adverse events induced in mice by intratracheal instillation of Milan PM1. This fraction represents PM with aerodynamic diameter ≤1 *μ*m [15], constituted by almost 40% of particles ≤400 nm in diameter. Lonati and Giugliano [16] monitored particles size distribution in four different sites in Milan and concluded that, at open air sites, 99.5% of the total number of particles are characterised by a diameter smaller than 1 *μ*m. 

## 2. Materials and Methods

### 2.1. Animals

Male BALB/c mice (7-8 weeks old) were purchased from Harlan Laboratories (Italy); food and water were administered *ad libitum.* Mice were housed in plastic cages under controlled environmental conditions (temperature 19–21°C, humidity 40–70%, lights on 7 am–7 pm). The established rules of animal care approved by Italian Ministry of Health (DL 116/92) were followed. Intratracheal instillations have been performed in mice under controlled general anaesthesia to avoid pain and discomfort. During the whole experiments we found no changes in mice weights or behaviour.

### 2.2. PM Sources and Chemical Characterization

Atmospheric PM1 was collected during 2007-2008 in a Milan urban area as described in previous papers [13]. The sampling site was located at Torre Sarca, an urban site with high vehicle traffic. Samplers were located in a fenced area at about 2.5 m from the ground, 10 m from the road, and 30 m from the nearest traffic light.

PM1 was sampled and chemical analyses were performed as described in Perrone et al. [17]; Milan PM1 chemical composition (inorganic ions, elements, and PAHs) is summarized in Table 1.

Particles' suspensions were prepared as follow: just before the intratracheal instillation, PM1 aliquots were properly diluted in sterile pyrogen-free saline, sonicated and vortexed, and then immediately instilled in mice.

### 2.3. Dose

Our study was designed to measure the systemic response to repeated PM1 exposure and test in an animal model the hypothesis that sustained PM1 exposure could exert cardiovascular dysfunctions. Similar investigations have been previously based on very high PM exposure rate in single or repeated intratracheal instillation [20–23].

Lung inflammation play a key role in enhancing the extrapulmonary translocation of particles [21]. So, we tested the threshold valid to lengthen the PM1 proinflammatory effects within lungs of our BALB/c mice, and it resulted higher than the estimated daily dose possibly deposited at the hot spot of lungs in worst pollution conditions. Ideally, *in vivo* studies should be performed with realistic dose levels, but, as already indicated for *in vitro *systems [24], short-term *in vivo* applications have some limitations, first of all the necessity to obtain measurable responses within few days.

We started from the dose used by Happo et al. [22], who instilled in mice a cumulative dose of 0.82 mg/animal of fine PM in a week, and we reduced the cumulative dose to 0.3 mg/animal of PM1 within the same time frame, in order to avoid particles lungs overload. The PM dose here used is not directly correlated to human urban PM exposures, but it has been determined as the lowest which induces a mild but still sustained lung inflammatory response in PM1 exposed mice.

### 2.4. Intratracheal PM1 Instillation and Broncho Alveolar Lavage

Animal testing was carried out by instilling 4 mice for each experimental group and the experiment was replicated twice, for a total of 8 sham and 8 PM1-treated mice.

Male BALB/c mice were briefly exposed to a mixture of 2.5% isoflurane (flurane) anesthetic gas and kept under anaesthesia during the whole instillation procedure. Once a deep stage of anaesthesia was reached, mice were intratracheally instilled by means of MicroSprayer Aerosolizer system (MicroSprayer Aerosolizer- Model IA-1C and FMJ-250 High Pressure Syringe, Penn Century, USA) with 100 *μ*g of PM1 in 100 *μ*L of isotonic saline solution, or 100 *μ*L of isotonic saline solution (sham) as previously reported [4, 10, 14]. Each mouse was placed in a supine position, the mouth was opened and the tongue was gently moved aside using a pince to better incannulate the trachea. The particles were suspended in the appropriate solution just before the intratracheal instillation. PM1-treated and sham mice were allowed to recover under visual control before placing them back in plastic cages, under controlled environmental conditions. The intratracheal instillation was performed on days 0, 3, and 6, for a total of three instillations. 24 h after the last instillation, mice from each experimental group were euthanized with an anesthetic mixture overdose (Tiletamine/Zolazepam-Xylazine and isoflurane). The effects were assessed 24 h after the last treatment since the greatest inflammatory response occurs by this time [22]. The broncho alveolar lavage (BAL) procedure, pellets, and supernatant recovery have been performed as described in Mantecca et al. [4, 14].

### 2.5. BALf Biochemical Assays

#### 2.5.1. Cell Counts

After centrifugation, the BALf pellets were resuspended in 500 *μ*L of DMEM (10% FBS, 1% penicillin-streptomycin, 1% glutamine), and total and differential cells counts performed as described in Mantecca et al. [4, 14].

#### 2.5.2. LDH and ALP

LDH and ALP assays were performed on cell-free BALf supernatants. The commercially available kits for alkaline phosphatase (DALP-250 QuantiChrom Alkaline Phosphatase Assay Kit, Gentaur Molecular) and lactate dehydrogenase (DLDH-100 QuantiChrom Lactate Dehydrogenase Kit, Gentaur Molecular) were employed according to the manufacturers' instructions. 

#### 2.5.3. Cytokines

The analyses of proinflammatory cytokines released in the BALf was performed by DuoSet ELISA kits for tumour necrosis factor-*α* and macrophage inflammatory protein-2 (TNF-*α* and MIP-2; R&D Systems) according to the manufacturer's protocols. 

### 2.6. Lung and Heart Biochemical Analyses

The lungs of sham and PM1-treated mice, at the end of BAL procedure, were quickly excised from the chest and washed in ice cold isotonic saline solution. The left lobes were dissected and submitted to histology, the right lobes were preserved for the biochemical analyses. For protein assays, lungs were minced at 4°C, suspended in NaCl 0.9%, briefly homogenized for 30 seconds at 11000 rpm with Ultra-Turrax T25 basic (IKA WERKE) and sonicated for other 30 seconds.

Then samples were submitted to trichloroacetic acid (TCA) precipitation according to the procedure described in Farina et al. [10]. The pellets were suspended in water and protein quantity determined by BCA method (Sigma Aldrich, USA). 

Thereafter, lung homogenates of sham and PM1-treated mice were loaded on SDS-PAGE and submitted to electrophoresis, followed by Western blot. The membranes were stained with Ponceau and the protein loading was assessed by densitometry (BIORAD Densitometry 710, program Quantity one) as described [25]. After blocking, blots were incubated for 2 h with the primary antibody diluted in PBS-Tween20/milk. Lung parenchyma was tested for Cyp1B1, HO-1, Caspase8-p18, NF*κ*B-p50, Hsp70, E-selectin, and iNOS (anti-Cyp1B1 1 : 200, anti-HO-1 1 : 200, anti-Casp8-p18 1 : 200, anti-NF*κ*B-p50 1 : 200, anti-Hsp70 1 : 200, anti-E-selectin 1 : 200, anti-iNOS 1 : 200, all by Santa Cruz). Then, blots were incubated for 1.5 h with horseradish peroxidase-conjugated anti-rabbit IgG (1 : 5000) or anti-goat IgG (1 : 2000) diluted in PBS-T/milk. 

Proteins were detected by ECL using the SuperSignal detection kit (Pierce, Rockford, IL). Immunoblot bands were analyzed and the optical density (OD) quantified by KODAK (Kodak Image Station 2000R); all the data have been normalized to *β*-actin (anti-*β*-actin 1 : 1500 by Sigma) and each protein in PM1-treated group was normalized to the corresponding sham group.

Heart tissue from sham and PM1-treated mice was submitted to all the procedures above described for lungs, and homogenates tested for HO-1, Caspase8-p18, NF*κ*B-p50, E-selectin, iNOS, and MPO (anti-HO-1 1 : 200, anti-Casp8-p18 1 : 200, anti-NF*κ*B-p50 1 : 200, anti-E-selectin 1 : 200, anti-iNOS 1 : 200, anti-MPO, all by Santa Cruz). 

### 2.7. Lung Histopathological Analyses

Once excised, the left lungs from sham and PM1 treated mice were fixed in Bouin's solution, embedded in paraffin, cross-sectioned at 7 *μ*m thickness by a rotary microtome, mounted on slides, and stained by hematoxilin and eosin (HE). Some sections were mounted onto Superfrost slides and processed for the immunohistochemical detection of HO-1, as previously reported [4], using a rabbit anti-HO-1 polyclonal antibody (Santa Cruz), and the peroxidase-based Vectastain Elite ABC Kit (Vectastain Laboratories) to visualize the immunochemical reaction. Slides were observed under a Zeiss Axioplan light microscope and images taken with a ZeissAxioCam MRc5 digital camera interfaced with the Axiovision Real 4.6 software.

### 2.8. Blood Analyses

Blood of sham and PM1-treated mice was collected by intracardiac puncture. Plasma has been recovered after two centrifugation, the first at 2000 g for 20 minutes and the second at 10000 g for 10 minutes at 4°C to completely remove platelets, and then submitted to sP-Selectin (Quantikine Mouse sP-selectin, R&D Systems), fibrinogen (Mouse Fibrinogen Antigen assay, Molecular Innovations), PAI-1 (murine PAI-1 activity assay, Molecular Innovations), and cytokines analyses (TNF-*α* and MIP-2; R&D Systems).

### 2.9. Statistical Analyses

Results are expressed as mean ± standard error of the mean (SE). Data distribution was tested by Shapiro-Wilk test; statistical differences were tested accordingly by *t*-test or non parametric *U* Mann-Whitney test. Statistical differences were considered to be significant at the 95% or 99% level (*P* < 0.05 or *P* < 0.01).

## 3. Results 

### 3.1. BALf Analyses

#### 3.1.1. Cell Counts

**Figure 1:**
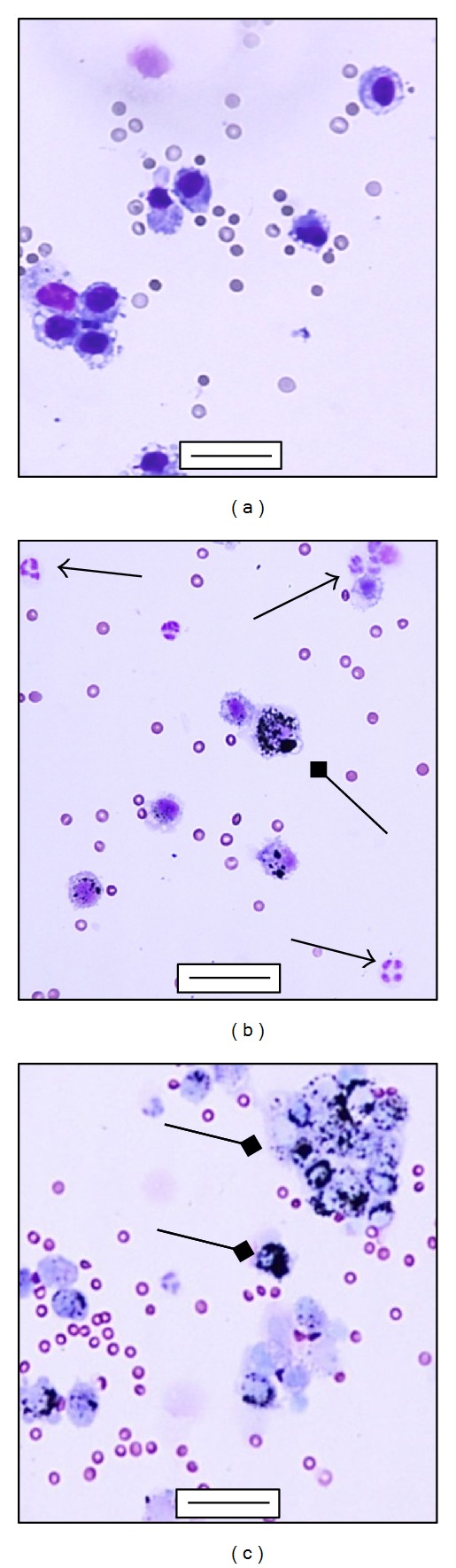
Differential staining of BALf cells. (a) Alveolar macrophages in the BALf collected 24 h postinstillation from sham; (b) and (c) alveolar macrophages engulfing particles (square arrows) and infiltration of PMNs (arrows) in the BALf collected 24 h after the last intratracheal instillation from PM1-treated mice. (a), (b), (c) bars = 50 *μ*m.

Significant increases of total cells number and lymphocytes (Ls) percentage (Table 2) have been found in PM1-treated mice. Polymorphonuclear cells (PMNs) percentage (sham 9.41 ± 2.75%, PM1-treated 14.27 ± 5.51%) as well as alveolar macrophages (AMs) percentage (sham 90.35 ± 2.79%; PM1-treated 84.72 ± 5.86%) were basically unaffected by PM1 treatment. However, AMs full of particles are clearly visible in the BALf of PM1-treated mice (Figures 1(a)–1(c)).

#### 3.1.2. LDH, ALP, and Cytokines

Lactate dehydrogenase (LDH) and alkaline phosphatase (ALP) activities significantly increased in the BALf of PM1-treated mice (Table 2). 

MIP-2 concentration increased in the BALf of PM1-treated mice (Table 2), while TNF-*α* was unchanged (sham 22.30 ± 6.89 pg/mL, PM1-treated 20.84 ± 6.57 pg/mL).

### 3.2. Lung Analyses

**Figure 2:**
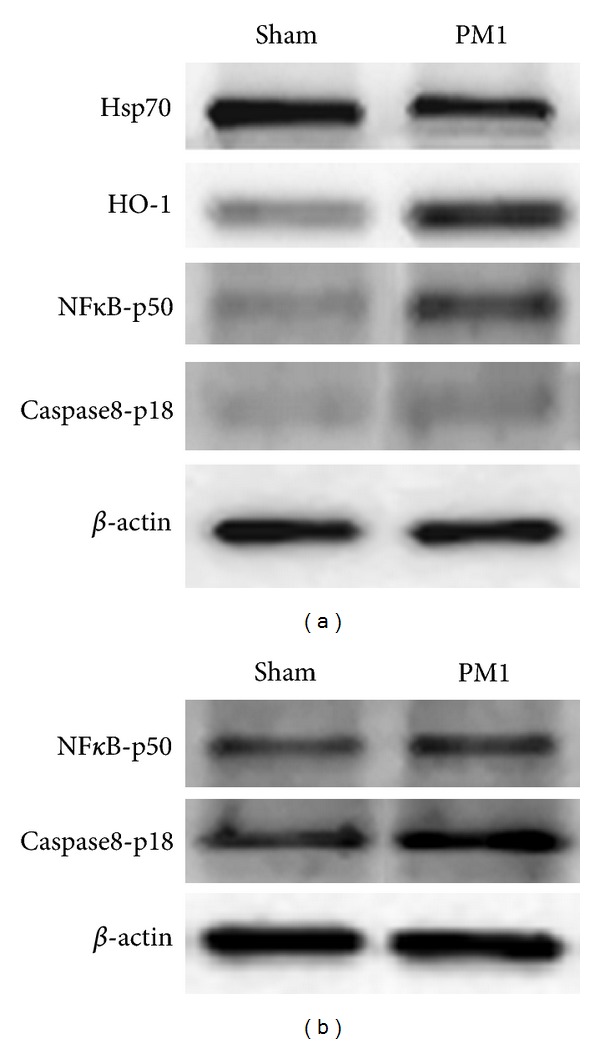
Protein analyses. (a) Representative western blottings showing Hsp70, HO-1, NF*κ*B-p50, Caspase8-p18 and *β*-actin in lung parenchyma in sham and PM1-treated mice. (b) Representative western blottings showing NF*κ*B-p50, Caspase8-p18 and *β*-actin in hearts of sham and PM1-treated mice.

**Figure 3:**
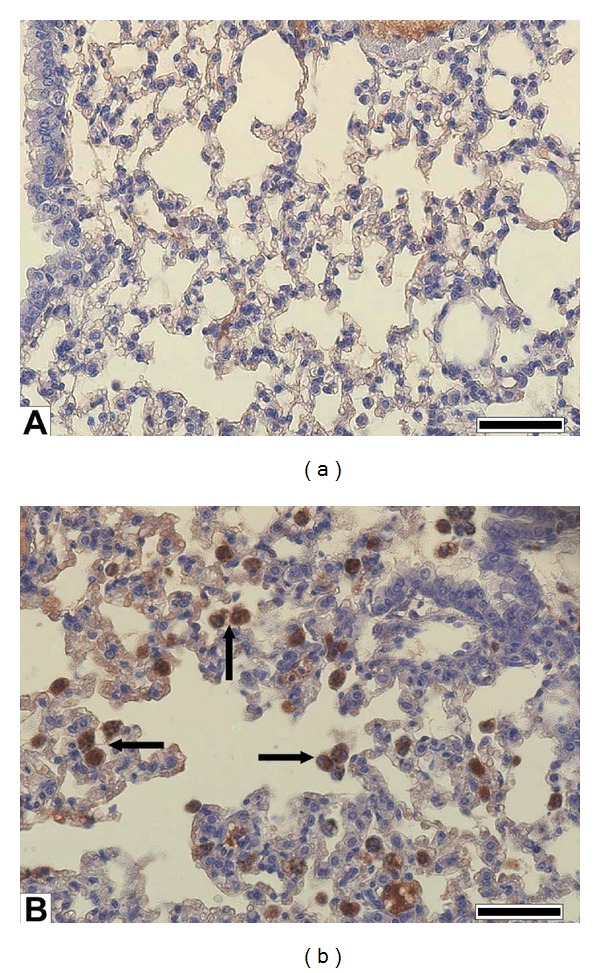
Lung parenchyma HO-1. Immunohistochemistry of HO-1 in lung tissues. Immunochemical reaction was developed by peroxidase and is visible as brown precipitate of DAB. (a) Sham lung showing no appreciable HO-1 signal; (b) PM1-treated lung showing intense HO-1 signal in particles' engulfed in alveolar macrophages (arrows). (a) and (b) bars = 50 *μ*m.

PM1 treatment induced a significant reduction of Hsp70 (heat shock protein 70, a functionally related protein involved in proteins folding) and a significant increase in HO-1 levels (heme oxigenase-1, a stress related protein which catalyzes heme degradation) in the lung parenchyma (Table 2 and Figure 2(a)), while iNOS (inducible nitric oxide synthase) was unchanged (sham 1 ± 0.35; PM1-treated 5.02 ± 0.52). The immunohistochemical analyses to evidence the HO-1 expression confirmed the activation of this antioxidant protective protein in the deep lung. HO-1 was mainly localized in AMs and in the alveolar epithelium (Figures 3(a) and 3(b)).

NF*κ*B rules the transcription of different genes, including pro- and antiapoptotic, and pro- and anti-inflammatory ones. A significant increase of its active fragment p50, as well as of the active fragment of the proapoptotic marker Caspase8-p18, was detected in the lungs of PM1-treated mice (Table 2 and Figure 2(a)).

Cyp1B1, a cytochrome of the P450 superfamily involved in the activation of many xenobiotics and in polycyclic aromatic hydrocarbons (PAHs) metabolism, did not increase in PM1-treated mice (sham 1 ± 0.12; PM1-treated 1.32 ± 0.07) as well as the E-selectin (sham 1 ± 0.21; PM1-treated 1.33 ± 0.18), a cell adhesion molecule related to inflammation. 

**Figure 4:**
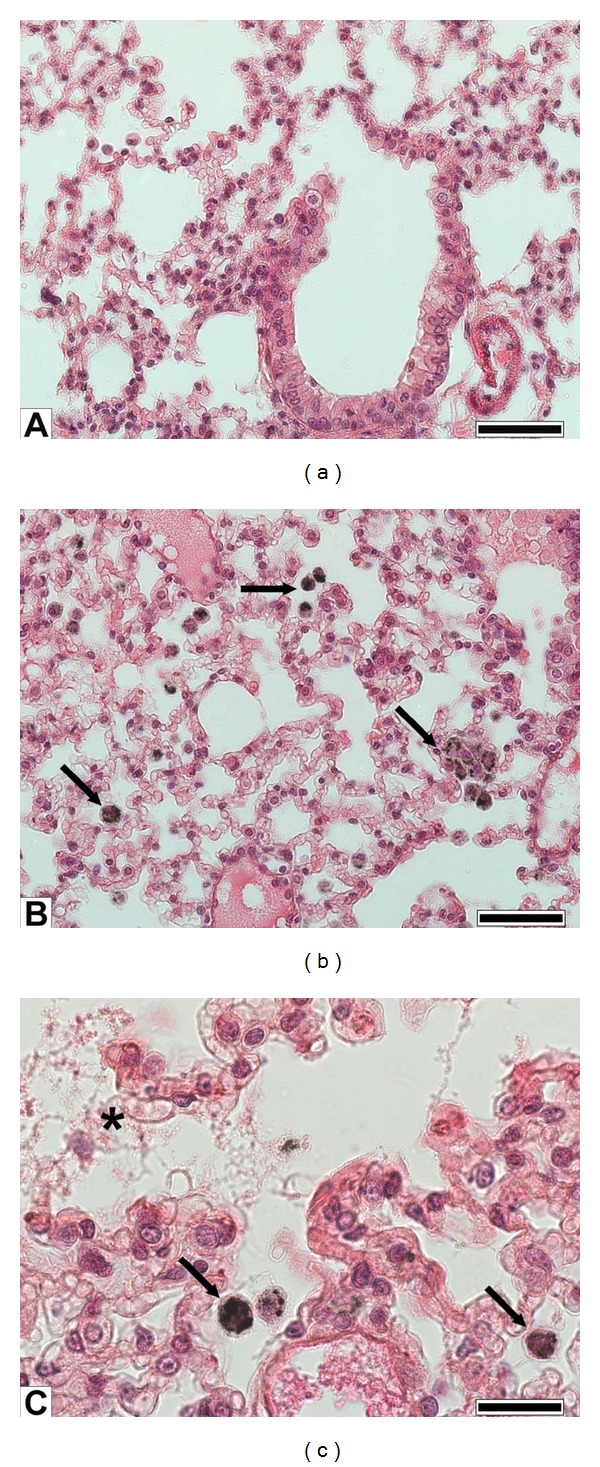
Lung histology. (a) Sham lung parenchyma showing bronchiolar and alveolar epithelia; (b) PM1-treated lung showing abundant alveolar macrophages engulfing particles (arrows); (c) detail of alveoli of a lung instilled with PM1 showing particles phagocytosis by alveolar macrophages and damage of the alveolar epithelium (asterisk). (a), (b) bars = 50 *μ*m; and (c) bar = 20 *μ*m.

Following PM1 exposure, 24 h after the last instillation, the histological evaluation of PM1-exposed lungs fail to disclose massive inflammation (Figures 4(a) and 4(b)). The most significant evidence in PM1 treated lungs was the ubiquitous presence in the alveolar airspace of AMs full of PM1 associated to lyses of the alveolar epithelium (Figures 4(b) and 4(c)). These data evidenced the active involvement of AMs in PM1 clearance and the direct cytotoxic effects elicited by PM1 on the lung alveolar epithelium, as confirmed by LDH, ALP, and Caspase-8 analyses.

### 3.3. Heart Analyses

MPO (myeloperoxidase, a marker of acute inflammation), iNOS, and E-selectin did not increase in heart of PM1-treated mice (MPO: sham 1 ± 0.16, PM1-treated 1.07 ± 0.15; iNOS: sham 1 ± 0.07, PM1-treated 0.95 ± 0.1; E-selectin: sham 1 ± 0.18, PM1-treated 0.94 ± 0.09). Consistent with these observations, 24 h after the last intratracheal instillation of PM1, HO-1 was basically unchanged (sham 1 ± 0.13; PM1-treated 1.05 ± 0.06), while NF*κ*B-p50 and Caspase8-p18 levels increased (Table 2 and Figure 2(b)).

### 3.4. Blood Analyses

Prothrombogenic and proinflammatory markers were analysed within the plasma of sham and PM1-treated mice: sP-selectin, a well-known marker of the activated platelet/endothelium interface, was significantly increased 24 h after the last intratracheal instillation in PM1-treated mice (Table 2). Fibrinogen (sham 2.89 ± 0.10 ng/mL, PM1-treated 2.91 ± 0.07 ng/mL) and PAI-1 plasma concentration (sham 0.15 ± 0.05 ng/mL, PM1-treated 0.23 ± 0.03 ng/mL), as well the cytokines MIP-2 and TNF-*α* (under kit detection limits, data not shown), were unaffected by PM1 intratracheal instillation.

## 4. Discussion

Our previous investigations [10] disclosed that a single instillation of fine particles in mice stimulates mild lung inflammation. The current study extends these findings, showing that repeated instillations of fine particulate matter trigger systemic adverse effect. The systemic response following repeated particle exposure could be due to a different pattern of the inflammatory mediators released from the lung, as compared with acute exposure. 

### 4.1. Inflammation and Injury in Mice Lungs

Increased BALf total cells counts in PM1-treated mice suggested a recruitment of proinflammatory cells in the alveolar spaces indeed, 24 h after the third instillation an increase in Ls percentage was clearly visible as well as AMs burdened by particles (Figures 1(a)–1(c)). 

In the BALf of healthy mice, AMs are abundant (>90%) while neutrophils are rare [26]. The PM1-intratracheal instillation could facilitate the deposition of particles in the alveolar spaces, where they come in contact with AMs, the first cells actively engaged in the clearance of inhaled particles [27]. Many studies have demonstrated that inhaled fine particles and aggregates of ultrafine particles are able to burden AMs thus impairing their phagocytosis [2, 28]. Such AMs in the BALf of treated mice could trigger lymphocellular inflammatory reaction within the bronchoalveolar districts. Following particles phagocytosis, AMs usually migrate to bronchoalveolar junctions, where they tend to accumulate and aggregate [29], releasing inflammatory mediators thus inducing a slight influx of neutrophils. 

An increase in the number of T lymphocytes has already been demonstrated in bronchial biopsies of healthy human volunteers exposed to PM [30]; moreover, PM has been shown to drive T-cell mediated cytokine production in the BALf of treated mice [31]. Many potentially biologically active components such as endotoxin, metals, polycyclic aromatic hydrocarbons (PAHs), and ozone might activate lymphocytes in the lung of PM-treated mice [23, 31]. PM1 induced changes in total and differential cells counts may be due both to a mild PMNs and Ls recruitment associated to reduced AMs migration toward the bloodstream. 

In our *in vivo* study no significant change in TNF-*α* concentration was evident in the BALf of PM1-treated mice, while MIP-2 concentration was significantly increased comparing to sham, suggesting that inflammation is still present 24 h after the third intratracheal instillation of PM1. 

However within the lungs, PM1 failed to induce the expression of proinflammatory adhesion molecules associated with endothelial activation, as confirmed by the E-selectin levels basically unchanged in sham and PM1-treated mice.

Fine and ultrafine particles have large surface area and therefore the adsorbed chemicals are largely bioavailable for redox or electrophilic chemistry [32]. It has been proposed that the proinflammatory process induced by particles could be related to the presence of PAHs [33]. Becker et al. [34] found that also Cr, Mn, Fe, Al, Si, Ti, and Cu may be related with cytokines production. Organic chemical components and transition metals associated with PM1 may thus contribute to adverse health effects based on their ability to induce oxidative stress responsible for the lung alveolar inflammation. 

Oxidative stress and proinflammatory cytokines are known to induce HO-1 expression in various cell types, including type II pneumocytes and AMs [35]. HO-1 acts as defence protein and its deficiency leads to enhanced endothelial cells injury [36]: the role of HO-1 is to catabolize the heme group from the cytosol, thus generating CO, biliverdin (converted to bilirubin) and Fe^2+^; all these products are thought to play a putative protective role against the inflammation onset and progression [37]. HO-1 increased in PM1-treated mice in agreement with previous data related to airborne pollutant toxicity both *in vivo* [4, 10, 38] and *in vitro* systems [39, 40], and could account for the mild ongoing lung inflammation we found in PM1-treated mice. Due to the role of HO-1 in regulating cellular heme availability for structural and functional heme-dependent proteins [41], within lungs no change in Cyp1B1 and iNOS levels were induced by PM1. 

AMs infiltrated in the lung parenchyma are positively stained for HO-1, thus suggesting that AMs in PM1-treated mice were suffering for oxidative stress due to the large burden of particles. Within the lungs, the pool of inflammatory phagocytes is the most significant and important cellular ROS generating system [42]; metals and organic substances adsorbed on PM surface have been related to their phagocytic oxidative burst [42].

Several transition metals adsorbed onto fine particles have been proved to trigger the generation of reactive oxygen species, in turn able to activate NF*κ*B, one of the most important mechanisms involved in PM induced pulmonary toxicity [43]. In our investigations, p50, one of the active subunits of the NF*κ*B transcription factor, increased in PM1-treated mice, in agreement with previous findings [10].

It has been reported [2, 44] that ultrafine particles, which are not efficiently cleared via mucociliary or macrophage-mediated mechanisms, very likely may enter the epithelial cells, cause injury to the integrity of the alveolar and endothelial cells thus spreading within the circulatory system. Increased LDH and ALP activity in the BALf of PM1-treated mice could be strictly related to the alveolar epithelium damage. Supporting this hypothesis and in agreement with previous investigations [4, 10, 14, 22, 41], histological analyses showed signs of alveolar cells damage within lungs of PM1-treated mice. Gerlofs-Nijland et al. [33] suggested that metals may contribute to the alveolar cell lyses and consequently to the LDH leakage in the BALf. In addition, the significant increase in Caspase-8 activation found in lung parenchyma of PM1-treated mice strengthens the hypothesis of a direct cell damage and apoptosis on AMs and lung epithelial cells mediated by fine particles [45].

It is generally known that HSPs are increased during cell stress. Surprisingly, Hsp70 levels in lung parenchyma of PM1-treated mice were significantly lower than in sham. Also, Stoeger et al. [46] reported reduced Hsp70 mRNA expression after particles instillation in BALB/c mice. Indeed, Hsp70 in the lungs is expressed by bronchial epithelium, alveolar cells and AMs [47]. HSPs may have a rapid turnover, especially during cell stress [48], and both the synthesis and chaperoning action of HSPs are energy requiring. Therefore, we might speculate that an energy imbalance and the increased turnover of lung epithelial cells, as demonstrated by high ALP activity in the BALf, did not permit the synthesis of sufficient quantities of Hsp70. 

Taking together, these results prove that PM1 promotes in instilled mice a mild ongoing lung inflammation in agreement with previous findings [32], thus triggering pro-oxidative and cytotoxic effects, both on AMs and lung cells. 

### 4.2. Effects on Mice Cardiovascular System

Epidemiological studies provided evidences of serious health hazards linked to human exposure within highly polluted urban centres PM [49]. 

Growing experimental evidences suggest that inhaled smallest particles can indeed translocate into the blood systemic circulation reaching extrapulmonary organs, such as heart and brain [50]. These adverse systemic effects might occur after fine or ultrafine particles inhalation basically in the absence of symptomatic and clinically detectable lung inflammation [32]. In our study, no variations in several inflammation and oxidative stress markers on heart tissues of PM1-treated mice were observed. However, an increase in NF*κ*B-p50 expression has been found in heart tissue, as already described after nanoparticles intratracheal instillation in rats [51].

Once again we observed increased activation of Caspase-8 in the hearts of PM1-treated mice, thus indicating the activation of the Caspase cascade. Cardiomyocytes apoptosis may be involved in the cardiac function impairment triggered by fine PM [52]. These findings are in agreement with the assumption that PM1 mainly exerts a direct cytotoxic effect on heart.

A direct correlation has been found between fine particles inhalation and increased fibrinogen level, plasma viscosity and red blood cell count [53]. Many data indicates the adhesion of platelets to the endothelium before the development of manifest atherosclerotic lesions [54]. Furthermore, is generally accepted that platelets contribute to the final stages of cardiovascular diseases, thus in thrombosis and myocardial infarction [55]. Soluble P-selectin (sP-selectin) is considered a marker of an activated platelet/vasculature/blood interface, as it can be released by activated platelets as well as by activated endothelial cells [53]. Indeed, we found a significant increase in sP-selectin concentration within plasma of PM1-treated mice, though both fibrinogen and PAI-1 concentration did not change and TNF-*α* and MIP-2 concentration were under the kit detection limits.

Among several hemostasis and inflammation mediators, only sP-selectin blood concentration was associated with preclinical cardiovascular risk, thus conferring to sP-selectin assay a clinical usefulness for detecting and managing high cardiovascular risk in primary prevention [56]. 

## 5. Conclusions

Short term exposure to PM1 induced in the lungs of BALB/c mice a mild inflammation, still ongoing 24 h after the last instillation. Particles escaping phagocytosis by impaired and overloaded macrophages could then elicit their cytotoxic effect directly on alveolar cells.

The inhaled PM1 could exert a progression of preexisting peripheral arterial occlusive disease sustaining the adhesion of platelets to the endothelium and considerably increasing thrombosis and myocardial infarction risks. 

A better understanding of mediators and mechanisms of these processes is mandatory if strategies have to be developed for individual protection to the PM-induced cardiovascular risk.

## Figures and Tables

**Table 1:** Table summarizing mean chemical composition (*μ*g/*μ*g PM) of 8 PM1 pooled samples (modified by “Seasonal variations in chemical composition and *in vitro* biological effects of fine PM from Milan” [17]). Inorganic ions explained about the 43% of the PM mass, the sum of all elements explained about the 0.8% while the contribution of PAHs was 0.016%. BaP: benzo[a]anthracene; BeP: benzo[e]pyrene; Bb+jF: benzo[b+j]fluoranthene; BkF: benzo(k)fluoranthene; BaP: benzo[a]pyrene; dBahA: dibenzo[a,h]anthracene; BghiP: benzo[g,h,i]perylene; IcdP: indeno[1,2-Cd]pyrene. Particles size distribution over the Milano metropolitan area has been studied by Ferrero and colleagues [18]. Concerning sources, traffic and heating during cold season constitute the 49–53% of the primary combustion sources of fine PM; during warm season they constitute about the 25%, while secondary sources are predominant (50–66%) [19]. Elemental carbon (primarily from traffic) contributes for about 10–15% to the fine fraction; organic matter, calculated applying a specific organic matter-to-organic carbon conversion factor to each source, contributes for 31–38% to the fine fraction [19].

Inorganic ions		Elements		PAHs	
****	Mean		Mean		Mean
F^−^	0.0001125	Al	0.000334	BaA	0.000011
Cl^−^	0.0061875	As	0.000025	Cr	0.000016
NO_3_^−^	0.1905875	Ba	0.000046	BeP	0.000025
PO_4_^3−^	0.001	Cd	0.00001	Bb+jF	0.000041
SO_4_^2−^	0.091325	Cr	0.00005	BkF	0.00001
Na^+^	0.0022375	Cu	0.000369	BaP	0.000017
NH_4_^+^	0.1348875	Fe	0.005804	dBahA	0.000001
K^+^	0.0062875	Mn	0.000081	BghiP	0.000022
Mg^2+^	0.0001125	Mo	0.000053	IcdP	0.000015
Ca^2+^	0.0012125	Ni	0.00005		
		Pb	0.000251		
		V	0.000025		
		Zn	0.00099		

**Table 2:** Table summarizing significant results in BALf, lung, heart, and blood in sham and PM1-treated mice, 24 h after the last intratracheal instillation. Concerning protein markers in lung and heart tissues, the data were normalized for the corresponding *β*-actin signal in each lane and expressed in relative to sham value. Data distribution was tested by Shapiro-Wilk test; statistical differences were tested by *t*-test or by non parametric *U* Mann-Whitney test. The data are expressed as mean ± SE. Sham versus PM1-treated: *P < 0.05; **P < 0.01.

		Sham		PM1		*P *
		Mean	±s.e.	Mean	±s.e.
BALf	Total cells (*E* + 05)	7.28	1.41	10.48	1.43	*
	% Ls	0.24	0.14	0.78	0.26	**
	LDH (IU/L)	19.49	1.59	27.5	0.4	**
	ALP (IU/L)	0.07	0.02	0.15	0.01	**
	MIP-2 (pg/mL)	58.99	9.52	102.12	12.12	*

Lung	Hsp70	1	0.04	0.75	0.03	*
	HO-1	1	0.04	5.28	0.97	*
	NF*κ*B-p50	1	0.18	3.12	0.13	*
	Casp8-p18	1	0.04	1.42	0.11	*

Heart	NF*κ*B-p50	1	0.02	1.37	0.08	*
	Casp8-p18	1	0.19	1.84	0.18	*

Blood	sP-selectin (ng/mL)	97.8	6.82	132.03	4.87	**
